# The interplay between iron accumulation, mitochondrial dysfunction, and inflammation during the execution step of neurodegenerative disorders

**DOI:** 10.3389/fphar.2014.00038

**Published:** 2014-03-10

**Authors:** Pamela J. Urrutia, Natalia P. Mena, Marco T. Núñez

**Affiliations:** Department of Biology and Research Ring on Oxidative Stress in the Nervous System, Faculty of Sciences, University of ChileSantiago, Chile

**Keywords:** inflammation, neurodegeneration, mitochondrial dysfunction, iron toxicity, Parkinson’s disease

## Abstract

A growing set of observations points to mitochondrial dysfunction, iron accumulation, oxidative damage and chronic inflammation as common pathognomonic signs of a number of neurodegenerative diseases that includes Alzheimer’s disease, Huntington disease, amyotrophic lateral sclerosis, Friedrich’s ataxia and Parkinson’s disease. Particularly relevant for neurodegenerative processes is the relationship between mitochondria and iron. The mitochondrion upholds the synthesis of iron–sulfur clusters and heme, the most abundant iron-containing prosthetic groups in a large variety of proteins, so a fraction of incoming iron must go through this organelle before reaching its final destination. In turn, the mitochondrial respiratory chain is the source of reactive oxygen species (ROS) derived from leaks in the electron transport chain. The co-existence of both iron and ROS in the secluded space of the mitochondrion makes this organelle particularly prone to hydroxyl radical-mediated damage. In addition, a connection between the loss of iron homeostasis and inflammation is starting to emerge; thus, inflammatory cytokines like TNF-alpha and IL-6 induce the synthesis of the divalent metal transporter 1 and promote iron accumulation in neurons and microglia. Here, we review the recent literature on mitochondrial iron homeostasis and the role of inflammation on mitochondria dysfunction and iron accumulation on the neurodegenerative process that lead to cell death in Parkinson’s disease. We also put forward the hypothesis that mitochondrial dysfunction, iron accumulation and inflammation are part of a synergistic self-feeding cycle that ends in apoptotic cell death, once the antioxidant cellular defense systems are finally overwhelmed.

## INTRODUCTION

Iron is an essential element necessary for the normal development of brain functions. Enzymes involved in neurotransmitter synthesis that possess iron as a prosthetic group are recognized targets of iron deficiency: monoamine oxidases A and B involved in dopamine catabolism, tryptophan hydroxylase, required for serotonin synthesis, tyrosine hydroxylase, required for dopamine and norepinephrine synthesis, glutamate decarboxylase involved in GABA synthesis and glutamate transaminase involved in L-glutamate synthesis, all belong to this group.

Abundant evidence shows that iron accumulation in particular areas of the brain is a hallmark of several neurodegenerative disorders (Zecca et al., 2004; Andersen et al., 2013), although it is uncertain whether iron accumulation is a primary cause of the disorder or a consequence of a previous dysfunction. Increased levels of iron promote cell death via hydroxyl radical formation, which enhances lipid peroxidation, protein aggregation, glutathione consumption, and nucleic acid modification. We recently put forward the hypothesis that iron accumulation, a process initiated by mitochondrial dysfunction, and the ensuing oxidative damage is part of the execution step, i.e., the death process of affected neurons (Núñez et al., 2012).

Mitochondrial dysfunction has long been associated with several neurodegenerative diseases that include Alzheimer’s disease (AD), Huntington’s disease (HD), Parkinson’s disease (PD), amyotrophic lateral sclerosis (ALS), and Friedrich’s Ataxia (FA; Schapira and Cooper, 1992; Moreira et al., 2010; Grubman et al., 2013). Mitochondrial dysfunction results in decreased ATP synthesis, as well as in decreased synthesis of iron–sulfur clusters (ISCs) and heme prosthetic groups. An association between mitochondrial dysfunction and mitochondrial iron accumulation has been found only in FA (Delatycki et al., 1999; Huang et al., 2009), although evidence for mitochondrial iron accumulation has been reported in experimental models of PD (Liang and Patel, 2004; Lee et al., 2009; Mena et al., 2011).

Inflammation in the central nervous system (CNS) is a condition strongly associated with neuronal death in several neurodegenerative disorders including PD and AD (Hirsch and Hunot, 2009). Inflammation is characterized by the occurrence of reactive microglia and a massive production of pro-inflammatory cytokines. These inflammatory processes trigger a chain of events including increased production of ROS and reactive nitrogen species (RNS), disruption of iron metabolism and mitochondrial dysfunction, finally leading to neurodegeneration.

## THE BASIS OF IRON TOXICITY

The ability of iron to exchange readily one electron underlies its insertion in numerous catalytic processes found in living matter. The iron atom has octahedral coordination chemistry; therefore, it has six possible coordination bonds. Seminal work by Graf and associates demonstrated that iron is redox-inactive only if all its six coordination sites are stably bound. If one of the sites is free or loosely bound, iron is redox-active and competent of undertaking one-electron exchange reactions (Graf et al., 1984). It is noteworthy that Fe^3^^+^ complexes with the chelators desferrioxamine, DTPA or phytate at 1:10 (mol:mol) ratio result in redox-inactive iron whereas Fe^3^^+^ chelation with NTA, EDTA, EGTA, ATP, CDTA or bleomycin results in redox-active iron at the same 1:10 molar ratio (Graf et al., 1984).

Iron is a paramagnetic element with two stable oxidation states: 2+ and 3+. As mentioned above, both Fe^2^^+^ and Fe^3^^+^ establish coordination complexes with a great variety of ligands. Iron complexes display a variety of reduction potentials, ranging from very positive to negative values because of a basic concept in coordination chemistry that establishes that the ligand modifies the electron cloud surrounding the metal, thus modifying its reduction potential. This versatility in reduction potential allows for fine-tuning between iron reduction potential and the electron transfer process it catalyzes. It is estimated that the predominant reduction potential for iron in the intracellular milieu of the cell is near zero V (Clark, 1960; Wood, 1988). Many *in vitro* experiments confirm iron-mediated production of the hydroxyl radical (^•^OH), which arises from the following reactions:

$$1.\;{Fe}^{2+}+O_{2}↔{Fe}^{3+}+O_{2}^{•‑}E_{0}:‑0.43V;\;\;\Delta G=41.5KJ/mol$$

$$2.\;O_{2}^{•‑}+{2H}^{+}\to H_{2}O_{2}+O_{2}E_{0}:0.55V;\;\;\Delta G=‑53.0KJ/mol$$

3. Fe2++H2O2→Fe3++OH+OH•  E0:0.10V; ΔG=‑9.7KJ/mol

The thermodynamic sum of reactions 1–3 gives reaction 4:

4. 3Fe2+2O2+2H+→Fe3++OH‑+OH• ΔG=‑21.2KJ/mol

The intracellular environment provides abundant reducing power in the form of GSH (mM) and Asc (μM), which reduces Fe^3^^+^ to Fe^2^^+^:

$$5.\;{Fe}^{3+}+GSH(Asc)\to {Fe}^{2+}GSSH\left(Asc•\right)+H^{+}E_{0}:0.262;\Delta G=‑25.3KJ/mol$$

Changes in free energy were calculated applying the equation *δG = *-*nFE*_0_ (Joule/mol), in which *n* is the number of electrons exchanged and *F* the Faraday constant. Reaction 1 values were from (Pierre and Fontecave, 1999); Reaction 2, the half-cell potential for H_2_O_2_ dismutation was considerer 0.45 V (Pierre and Fontecave, 1999) and the reduction potential of the Fe^3^^+^/Fe^2^^+^ half-cell was considered 0 V (Wood, 1988); Reaction 3 (Fenton reaction): *E*_0_ half-cell values from (Buettner, 1993; Buettner and Schafer, 2000). Half-cell potentials for reaction 5 were obtained from (Millis et al., 1993; Pierre and Fontecave, 1999). GSH: reduced glutathione; GSSG: oxidized glutathione; Asc: ascorbate; Asc•: ascorbate free radical.

The hydroxyl radical is considered one of the most reactive species generated in biological systems, since its reaction rate is only limited by diffusion, with rate constants in the 10^9^–10^12^ Mol^-^^1^ s^-^^1^ range (Davies, 2005). This molecule induces irreversible damage to DNA, RNA, proteins, and lipids. Indeed, the hydroxyl radical is believed to be the etiological agent for several diseases and may be involved in the natural process of aging (Lipinski, 2011).

The main components of cell iron homeostasis are the divalent metal transporter 1 (DMT1), a Fe^2^^+^ transporter that brings iron into the cell, the transferrin receptor 1 (TfR1) that brings iron in through the endocytosis of Ferro-transferrin, the iron export transporter ferroportin 1 (FPN1) and the cytosolic iron storage protein ferritin. The expression of these proteins is transductionally regulated by the iron responsive element/iron regulatory protein (IRE/IRP) system, which is activated when cells have low iron levels, resulting in increased DMT1 and TfR1 levels and decreased FPN1 and ferritin expression (Muckenthaler et al., 2008).

In cells, iron in the 0.2–1.5 μM range is weakly complexed to low-molecular weight substrates such as citrate, carboxylates, amines, phosphate, nucleotides, GSH, and other molecules conforming the “cytosolic labile iron pool” (cLIP; Epsztejn et al., 1997; Kakhlon and Cabantchik, 2002; Petrat et al., 2002; Hider and Kong, 2011). Iron in this pool is redox-active, cycling between the Fe^+^^2^ and Fe^+^^3^ forms, with prevalence of the reduced form because of the reductive cytosol environment. This redox-active pool is suitable to experimental detection by the fluorophore calcein, which has higher affinity for Fe^3^^+^ than for Fe^2^^+^ but since the reduction potential for iron in the Fe-calcein complex is low, Fe^3^^+^ bound to calcein is readily reduced in the intracellular environment, resulting in decreased calcein fluorescence (Petrat et al., 2002). In cultured neuroblastoma cells the LIP represents about 3% of total cellular iron under basal culture conditions, but this percentage increases 3–4 fold, to μM concentrations, after exposure of cells to high extracellular iron concentrations (Núñez-Millacura et al., 2002; Núñez et al., 2004). In cell models, iron overload generates increased lipid peroxidation, protein modifications and damage to DNA, consistent with the production of the hydroxyl radical (Mello-Filho and Meneghini, 1991; Núñez et al., 2001; Sochaski et al., 2002; Zoccarato et al., 2005).

## INFLAMMATORY CYTOKINES INDUCE THE PRODUCTION OF RNS, ROS AND IRON ACUMULATION

Postmortem tissues from patients with AD, PD, HD, ALS or FA show oxidative damage in the affected brain regions (Nunomura et al., 1999; Barnham et al., 2004; Emerit et al., 2004). The association between inflammation and oxidative damage is mediated by the release of RNS and ROS during the inflammatory process. In particular, activated microglia have high levels of nitric oxide synthase (NOS) and NADPH oxidase (NOX), two enzyme systems that mediate the increase in the oxidative tone induced by inflammation.

Microglia, the brain-resident immune cells, are essential for the generation of the inflammatory response. They are activated by distress signals released from neighboring cells, initiating an innate response characterized by the production of pro-inflammatory cytokines and, incidentally, phagocytosis (McGeer et al., 1988; Colton and Wilcock, 2010). Indeed, many cases of AD and PD are accompanied by a dramatic proliferation of reactive amoeboid macrophages and activated microglia in the substantia nigra (SN) or frontal cortex (McGeer et al., 1988; Possel et al., 2000; Kiyota et al., 2009; Hewett and Hewett, 2012), together with high expression of pro-inflammatory cytokines (Bauer et al., 1991; Mogi et al., 1994; Muller et al., 1998; Nagatsu, 2002; Hewett and Hewett, 2012).

Inducible NOS (iNOS, also called NOS-2), which is scarcely expressed in the brain is induced during gliosis in pathological situations including AD (Aliev et al., 2009) and PD (Dawson and Dawson, 1998). Up-regulation of iNOS and of cyclo-oxygenase-1 and cyclo-oxygenase-2 in amoeboid microglia occurs in the SN of human PD patients (Knott et al., 2000). A study on the levels of iNOS mRNA in postmortem PD basal ganglia found a significant increase in iNOS expression in the dorsal two-thirds of the striatum and in the medial medullary lamina of the globus pallidus, accompanied by a reduction in iNOS mRNA expression in the putamen (Eve et al., 1998).

Inflammatory mediators, including LPS and some cytokines (TNF-α, IL-1β, and IFN-γ) induce the transcriptional activation of the iNOS gene in astrocytes and microglia via activation of the transcription factors STAT1 and NF-kB (Grzybicki et al., 1996; Possel et al., 2000; Hewett and Hewett, 2012). These factors translocate to the nucleus and bind to response elements present in the iNOS coding sequence. Upregulation of microglial iNOS expression is also observed after administration of 1-methyl-4-phenyl-1,2,3,6-tetrahydropyridine (MPTP; Liberatore et al., 1999; Tieu et al., 2003; Kokovay and Cunningham, 2005; Yokoyama et al., 2008). Interestingly, administration of MPTP produces significantly less neuronal loss in mice deficient in iNOS compared to their wild type counterparts (Dexter et al., 1986; Liberatore et al., 1999; Dehmer et al., 2000). In the 6-hydroxidopamine (6-OHDA) model, iNOS activity in the striatum induces neurodegeneration in rats. Pretreatment with the iNOS inhibitor L-NAME blocks amphetamine-induced rotations and significantly restores striatal dopamine (DA) levels in 6-OHDA treated rats (Barthwal et al., 2001). In neuroinflammatory models of PD, iNOS also participates in nigral neurodegeneration. Injection of LPS induces iNOS expression in the SN in a time- and dose-dependent manner; iNOS is present mainly in fully activated microglia with the characteristic amoeboid morphology. Furthermore, LPS-induced loss of dopaminergic neurons decreases significantly by administration of an iNOS inhibitor (Arimoto and Bing, 2003; Singh et al., 2005).

The iNOS enzyme is a relevant factor in the neurodegenerative process associated to AD. Early observations reported increased iNOS and nitrotyrosine protein modifications in AD brains, mainly in neurofibrillary tangle-bearing neurons and neuropil threads as well as in astrocytes (Vodovotz et al., 1996; Smith et al., 1997; Wallace et al., 1997). Studies in transgenic mice overexpressing amyloid beta precursor protein (APP) demonstrated that several pathological changes such as vessel lesions, amyloid deposition and mitochondrial DNA deletions, are associated with the degree of NOS overexpression (Seyidova et al., 2004). Nevertheless, the APPsw/iNOS(-/-) mice, which express human APP mutations on an iNOS knockout background, show increased appearance of tau pathology, neuronal death, neuroinflammation and behavioral deficits compared with the parental APPsw mice (Colton et al., 2008). This evidence indicates that in AD, the production of NO can be protective or damaging, depending on the levels of NO production.

The phagocyte NOX is the main regulated source of ROS generation. The catalytic component of the NOX complex is composed by a family of multiple-pass transmembrane proteins, named NOX1–4. The most studied, NOX2, also known as gp91phox or phagocyte oxidase (PHOX), is highly expressed in innate immune cells including microglia and it is most likely the predominant NOX isoform expressed in astrocytes, while neurons express both NOX2 and NOX4 (Skalnik et al., 1991; Noh and Koh, 2000; Lavigne et al., 2001; Abramov et al., 2004; Pawate et al., 2004). NOX2 forms a complex with p67phox, p47phox, p40phox, and p22phox subunits. Several stimuli induce NOX2 complex priming, including pro-inflammatory cytokines (TNF-α, IL-1β) and Toll-like receptor (TLR) agonists like LPS, peroxynitrite and proteases. The primed NOX2 complex requires yet additional activation to initiate substantial ROS production. PKC activators, growth factors, complement protein C5a and G protein-coupled receptor agonists generate a fully active NOX complex (Yang et al., 2009,^2013^; Sareila et al., 2011).

Activation of NOX also occurs in experimental models of PD and AD. Treatment with MPTP results in increased synthesis of the proinflammatory cytokine IL-1β and increased membrane translocation of p67phox that is prevented by minocycline, a tetracycline derivative that exerts multiple anti-inflammatory effects (Wu et al., 2002). In addition, aging mice treated with MPTP display an increase in gp91phox and 3-nitrotyrosine (L’Episcopo et al., 2010; Huh et al., 2011). In agreement, gp91phox-/- mice display decreased degeneration of dopaminergic neurons induced by MPTP compared to wild type mice (Wu et al., 2003; Zhang et al., 2004). The unilateral injection of 6-OHDA into the right striatum of rats induces an increase of NOX1 and NOX2 both in the striatum and the SN. In concordance, dopaminergic neuronal and TNF-α and IFN-γ induction triggered by 6-OHDA are abrogated in the gp91phox-/- or minocycline treated mice (Hernandes et al., 2013). Additionally, striatal injection of 6-OHDA increases NOX1 expression in dopaminergic neurons in rat SN, and also increases 8-oxo-dG content, a marker of DNA oxidative damage. Moreover, NOX1 knockdown reduces 6-OHDA-induced oxidative DNA damage and dopaminergic neuronal degeneration (Choi et al., 2012).

Microglia of AD subjects display activated NOX2, resulting in the formation of ROS that are toxic to neighboring neurons (Shimohama et al., 2000). In conjunction, an increment in NOX1 and NOX3 mRNA levels in the frontal lobe tissue from AD brains was reported, suggesting the participation of other NOX family members in AD neuropathology (de la Monte and Wands, 2006). Recently, increased NOX-dependent ROS production in the superior/middle temporal gyri at the earliest clinical manifestations of disease, but not in late-stage AD, was reported (Bruce-Keller et al., 2010). Genetic inactivation of NOX2 in 12- to 15-month-old mice overexpressing the APPsw mutation (Tg2576 mice) results in reduced oxidative damage and rescues both the vascular and behavioral alterations observed in Tg2576 mice (Park et al., 2008). Studies done in cell cultures replicated the postmortem and animal findings on oxidative damage driven by NOX activation. Experiments using co-cultures of neuronal and glial cells found that Aβ acts preferentially on astrocytes but causes neuronal death (Abramov et al., 2004; Abramov and Duchen, 2005). The Aβ peptide causes transient increases in cytoplasmic calcium in astrocytes, associated with increased ROS generation, glutathione depletion and mitochondrial depolarization. Neuronal death after Aβ exposure was reduced both by NOX inhibitors and in the gp91phox knockout mice. These data are consistent with a sequence of events in which Aβ activates NOX in astrocytes by increasing cytoplasmic calcium, generating an oxidative burst that causes the death of neighboring neurons (Abramov et al., 2004; Abramov and Duchen, 2005; Park et al., 2008).

Inflammatory conditions such as those found in neurodegenerative diseases also affect iron homeostasis through transcriptional modification of iron transporters. In this context, the observation that the transcription factor NFκB induces DMT1 expression is highly relevant for understanding the relationship between inflammation and iron homeostasis (Paradkar and Roth, 2006). We recently reported that the pro-inflammatory cytokines TNF-α, IL-6 and the TLR4 agonist LPS directly regulate DMT1mRNA and protein levels and induce a transient decrease in FPN1 protein, thus generating an increment of iron content in neurons and microglia (Urrutia et al., 2013). Supporting the results described above, a recent study using primary cultures of ventral mesencephalic neurons demonstrated that TNF-α or IL-1β induce an increment in DMT1 and TfR1 protein levels, together with a reduction of FPN1 levels, resulting in an increase in ferrous iron influx and decreased iron efflux in neurons (Wang et al., 2013). These findings were replicated in systemic tissues. Treatment of mouse splenocyte with LPS down-regulates the expression of FPN1 through a signaling mechanism mediated by TLR4 (Yang et al., 2002). Moreover, stimulation of macrophage cell lines with IFN-γ, TNF-α or LPS results in increased IRE-binding activity of IRP1 and IRP2, and increased DMT1 mRNA expression (Mulero and Brock, 1999; Wardrop and Richardson, 2000; Ludwiczek et al., 2003; Wang et al., 2005).

Considering that NFκB activation takes place downstream of TNF-α, IL-1 and LPS signaling pathways (Teeuwsen et al., 1991; Rothwell and Luheshi, 2000; Hanke and Kielian, 2011), inflammatory stimuli may induce DMT1 expression via NFκB activation. Indeed, TNF-α was detected in glial cells in the SN of PD patients but not in control subjects, together with immunoreactivity for TNF-α receptors in dopaminergic neurons of both control and PD patients (Boka et al., 1994). These findings are suggestive of a circuit in which activation of nigral microglia results in TNF-α secretion, which might increase iron uptake by dopaminergic neuron via NF-κB-induced DMT1 expression. Indeed, an increase in the nuclear immunoreactivity of NFκB has been observed in PD brains or in animal models for this disease (Hunot et al., 1997), so it is possible that activation of NF-κB via inflammatory stimuli contributes to iron accumulation in PD. Accordingly, inflammation could induce the production of hydroxyl radical trough the activation of two parallel pathways: (i) through DMT1-mediated increase of intracellular iron levels and (ii) through increased hydrogen peroxide levels mediated by NOX activation.

A positive feedback loop can be established between ROS/RNS and inflammatory cytokines. ROS induce intracellular signaling pathways that result in the activation of transcriptional factors like NF-kB, AP-1 and Nrf-2, which regulate the expression of pro-inflammatory mediators such as Cox-2, MCP-1, IL-6, TNF-α, IL-1α, and IL-1β (Hensley et al., 2000; Thannickal and Fanburg, 2000; Ueda et al., 2002; Ridder and Schwaninger, 2009; Kitazawa et al., 2011; Guo et al., 2012; Kawamoto et al., 2012; Phani et al., 2012; Song et al., 2012; Zhang et al., 2012; Tobon-Velasco et al., 2013). These cytokines and chemokines, in turn, stimulate a cascade of events leading to increased oxidative stress via iNOS and NOX activation.

## INFLAMMATORY CONDITIONS INDUCE MITOCHONDRIAL DYSFUNCTION

The study of the relationship between inflammation and mitochondrial activity in the CNS is incipient. Intrastriatal injection of LPS induces mitochondrial dysfunction, microgliosis, iron accumulation and progressive degeneration of the dopamine nigro-striatal system (Zhang et al., 2005; Hunter et al., 2007, 2008; Choi et al., 2009), as observed in PD pathology. Similarly, cytokines such as IL-1β decrease mitochondrial activity through the production of NO in cardiomyocytes (Tatsumi et al., 2000).

Several reports indicate that TLRs regulate mitochondrial activity. Activation of TLR3 results in reduction of mitochondrial oxygen consumption mediated by opening of the permeability transition pore (Djafarzadeh et al., 2011). In co-cultures of cortical neurons with microglial cells, the TLR4 agonist LPS promotes decreased oxygen consumption and oxidative stress, with the subsequent nigral dopaminergic neuronal death in a rat model of inflammation (Xie et al., 2004; Hunter et al., 2007). Although these studies strongly suggest a link between TLRs and mitochondria dysfunction, further studies should clarify the molecular mechanisms involved and its relevance to particular neurodegenerative processes.

The production of ROS and RNS affects mitochondrial activity through destabilization of the ISCs (Cassina and Radi, 1996; Brown and Borutaite, 2004). The free radical superoxide damages and/or oxidizes 4Fe-4S clusters, which results in the formation of the “null” 3Fe-4S center form (Flint et al., 1993; Hausladen and Fridovich, 1994; Gardner et al., 1995; Bouton et al., 1996). Additionally, NO reacts with 4Fe-4S clusters generating [(NO)_2_Fe(SR)_2_] type complexes that inactivate several mitochondrial iron–sulfur enzymes including proteins which compose the electron transport chain (Drapier, 1997; see below). The above data are consistent with the notion that inflammation, ROS/RNS production, and mitochondrial dysfunction are linked processes.

Additionally, recent evidence shows that under certain conditions mitochondria can modulate the immune response. The mitochondrial protein MARCH5 (an ubiquitin E3 ligase constitutively expressed in the mitochondrion outer membrane) positively regulates TLR7 and TLR4 signaling, resulting in NFκB activation and expression of the NFκB-responsive genes IL-6 and TNF-α (Shi et al., 2011). In addition, activation of TLR1, TLR2 and TLR4 results in augmented mitochondrial ROS production by inducing translocation to mitochondria of TRAF6 (TLR signaling adaptor, tumor necrosis factor receptor-associated factor 6), which leads to the engagement and ubiquitination of ECSIT (evolutionarily conserved signaling intermediate in Toll pathways), a protein required for efficient assembly of mitochondrial complex I (West et al., 2011). It remains to be demonstrated whether this mechanism is operative in CNS cells.

Interestingly, mitochondrial ROS (mtROS) could arguably activate the inflammatory response. In vascular endothelium, mtROS act as intermediate signaling molecules to trigger production of IL-6 (Lee et al., 2010). In addition, patients with the autoinflammatory disorder TRAPS (tumor necrosis factor receptor-associated periodic syndrome), exhibit altered mitochondrial function with enhanced mtROS generation and increased production of IL-6, TNFα, and IL-1β; decreasing mtROS levels by the general antioxidant *N*-acetylcysteine effectively reduces inflammatory cytokine production after LPS stimulation (Bulua et al., 2011). These results point to novel pathways that link inflammation to mtROS production.

**FIGURE 1:**
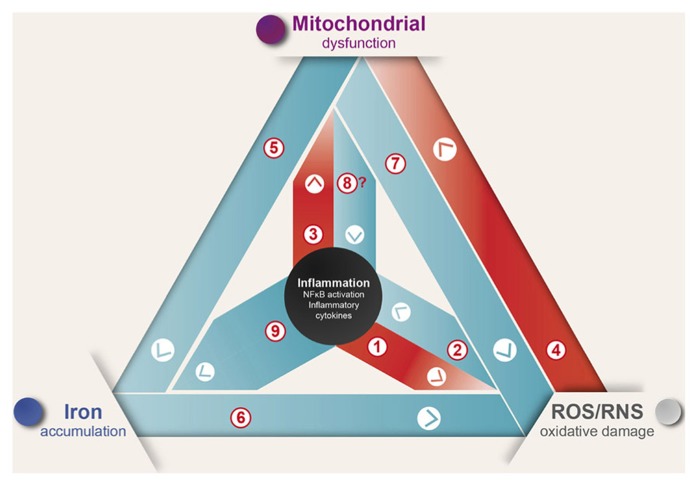
**Inflammation causes ROS/RNS production, mitochondrial dysfunction, and iron accumulation.** Inflammation, oxidative damage, and mitochondrial dysfunction are common features of neurodegenerative diseases. A complex net of relationships connect these features, which through feedback mechanisms contribute to the evolvement of neuronal death (see text for details).

In summary, inflammation induces ROS production and mitochondrial dysfunction generating a self-feeding cycle that could lead to neurodegeneration in diseases where inflammation and oxidative damage are prevalent (**Figure 1**). In this cycle, [1] inflammation induces ROS and RNS generation by activation of the NOX and iNOS enzymes (Possel et al., 2000; Sareila et al., 2011; Hewett and Hewett, 2012); [2] in turn, ROS/RNS induce the expression of inflammatory cytokines (Baeuerle and Henkel, 1994; Sen and Packer, 1996). [3] Additionally, inflammation induces mitochondrial dysfunction through activation of TLR signaling (Xie et al., 2004; Djafarzadeh et al., 2011). [4] ROS in turn induce mitochondrial dysfunction by destabilizing ISCs, which results in the inactivation of several mitochondrial iron–sulfur enzymes (Cassina and Radi, 1996; Brown and Borutaite, 2004). [5] Mitochondrial dysfunction leads to IRP1 activation and increased iron uptake (Lee et al., 2009; Mena et al., 2011). [6] Iron increases oxidative damage by transforming mild oxidative molecules like superoxide and hydrogen peroxide into the hydroxyl radical (Graf et al., 1984). [7] Electron transport chain inhibition increases ROS production by electron leak (Drose and Brandt, 2012), and arguably could modulate the innate immune response by TLR signaling regulation (Shi et al., 2011) [8]. Finally, [9] inflammation is likely to cause iron accumulation through induction of DMT1 expression and transient ferroportin decrease (Urrutia et al., 2013; Wang et al., 2013).

## MITOCHONDRIAL DYSFUNCTION, INFLAMMATION AND IRON ACCUMULATION IN THE DEATH OF NEURONS IN PD

Mitochondria have a key role in iron metabolism in association with the synthesis of ISCs and heme, prosthetic groups that are vital for cell function. Iron complexes are particularly relevant components of the electron transport chain: 12 proteins contain ISCs and eight proteins contain heme in their active centers (Rouault and Tong, 2005). Other proteins that have ISCs are the Krebs cycle enzymes aconitase and succinate dehydrogenase, ribonucleotide reductase, an enzyme that catalyzes the formation of deoxyribonucleotides from ribonucleotides, and ferrochelatase, involved in the addition of Fe to porphyrin IX during heme synthesis. We refer the reader to  for a comprehensive listing of ISC-containing proteins. Particular attention should be given to cytoplasmic IRP1, which contain a 4Fe-4S cluster in its inactive form and becomes active in the clusterless form (Haile et al., 1992; Shand and Volz, 2013).

Mitochondria have a redox-active iron pool (Petrat et al., 2001); an increase in this pool directly associates with an increase in oxidative damage and with calcium-dependent changes in the mitochondrial permeability transition pore (Pelizzoni et al., 2011; Kumfu et al., 2012; Zhang and Lemasters, 2013). Thus, cells must regulate tightly their mitochondrial Fe levels because an iron shortage affects numerous processes that have iron as a co-factor, including the electron transport chain, whereas an excess of redox-active iron promotes the generation of the noxious hydroxyl radical. How mitochondria regulate their iron content and what, if any, is the interplay between cytoplasmic and mitochondrial iron are incipient but highly relevant subjects to understand the mechanisms of mitochondrial dysfunction in neurodegenerative diseases.

There is increasing evidence that mitochondrial dysfunction plays an important role in the development of neurodegenerative diseases such as AD, HD, FA, and PD (Enns, 2003; Mandemakers et al., 2007; Sas et al., 2007; Gogvadze et al., 2009; Jellinger, 2009). Imbalances in ROS and ATP levels derived from mitochondrial dysfunction affect neurons particularly, given their dependence on ATP to propagate electrical signals, maintain ionic gradients, and facilitate anterograde and retrograde transport along axons (Su et al., 2013). The involvement of mitochondrial dysfunction in the pathophysiology of PD was noted very early in the study of the disease. Evidence of mitochondrial dysfunction in PD began in the eighties, when, after an intravenous injection of illicit drugs, four college students developed marked Parkinsonism. Analysis of the substances injected revealed the presence of MPTP, a compound metabolized by astrocytes into 1-methyl-4-phenylpyridinium (MPP+), which is then released into the extracellular space. MPP+ is taken up selectively by dopaminergic (DA) neurons where it inhibits mitochondrial complex I (Heikkila et al., 1984; Langston et al., 1984; Nicklas et al., 1985; Gautier et al., 2013). Further evidence showed that complex I activity and the number of complex I subunits are decreased in postmortem tissue of idiopathic PD patients (Bindoff et al., 1989; Mizuno et al., 1989; Schapira et al., 1989). These results strongly suggest that mitochondrial dysfunction is a pathognomonic sign in the pathophysiology of PD. Reduced complex-I activity and an increased susceptibility to MPP+ were also observed in cybrids containing mitochondrial DNA from PD patients (Swerdlow et al., 1996, 2001; Gu et al., 1998a), suggesting the presence of mitochondrial DNA-encoded defects in PD (Chaturvedi and Flint Beal, 2013). Additionally, in the epidemiology field, the use in farming of the highly lipophilic pesticide rotenone, a potent inhibitor of mitochondrial complex I, has been linked to a higher incidence of PD in agricultural workers (Betarbet et al., 2000; Tanner et al., 2011; Pezzoli and Cereda, 2013).

Mitochondrial complex I is a major source of ROS. Complex I from mitochondria of PD patients contain 47% more protein carbonyls localized to catalytic subunits and a 34% decrease in complex I 8-kDa subunit. NADH-driven electron transfer rates through complex I inversely correlate with complex I protein oxidation status and with the reduction in the 8-kDa subunit protein levels (Keeney et al., 2006).

Knowledge on the mechanisms that associate mitochondrial dysfunction and iron dyshomeostasis in PD is incipient. Treatment of SH-SY5Y dopaminergic neuroblastoma cells with mitochondrial complex I inhibitors such as rotenone or MPP+ results in ROS production and increased mitochondrial iron uptake (Lee et al., 2009; Mena et al., 2011). Moreover, inhibition of complex I by rotenone decreases the activity of three ISC-containing enzymes: mitochondrial and cytoplasmic aconitases and xanthine oxidase, and decreases the ISC content of glutamine phosphoribosyl pyrophosphate amidotransferase (Mena et al., 2011). The reduction in cytoplasmic aconitase activity is associated with an increase in iron regulatory IRP1 mRNA binding activity and with an increase in the mitochondrial labile iron pool (Mena et al., 2011). Since IRP1 activity post-transcriptionally regulates the expression of iron import proteins, ISC synthesis inhibition may result in a false iron deficiency signal with the ensuing iron accumulation.

**FIGURE 2:**
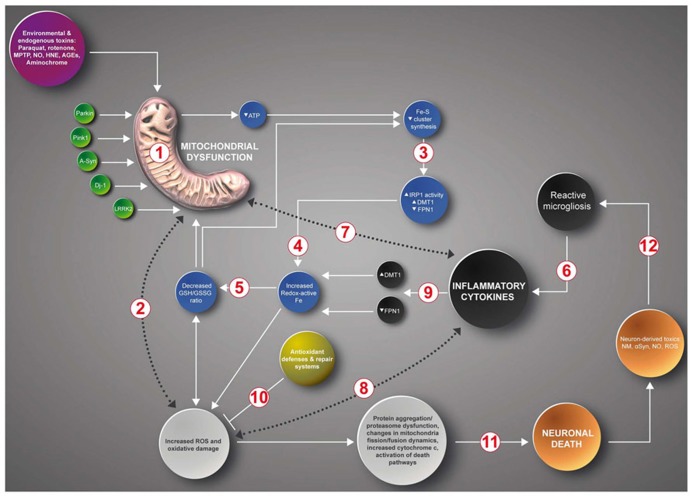
**A positive feedback loop in the death of neurons in PD.** Inhibition of mitochondrial complex I by endogenous or exogenous toxins or mutations in PD genes Parkin, Pink 1, Alpha-synuclein, DJ-1 or LRRK2 generates a multifactorial positive feedback loop. In this loop, complex I inhibition results in iron accumulation driven by decreased Fe-S cluster synthesis, IRP1 activation, increased DMT1 and TfR1 expression and decreased FPN1 expression, increased ROS levels and decreased glutathione levels. Both increased oxidative stress and low GSH levels further inhibit complex I activity. Another input to this cycle is contributed by inflammatory cytokines that through self-feeding cycles induce mitochondrial dysfunction, increased ROS/RNS production and iron accumulation mediated by the transcriptional regulation of DMT1 and FPN1 (see text). The cumulative oxidative damage finally results in apoptotic death (see text for details).

Considering the evidence discussed, we propose that inhibition of mitochondrial complex I by endogenous and/or exogenous toxins or by inflammatory processes resulting from trauma or other causes, engage a vicious cycle of increased oxidative stress and increased iron accumulation (**Figure 2**). In this scheme, inhibition of mitochondrial complex I by endogenous or exogenous toxins, or because of mutations in PD genes Parkin, Pink 1, alpha-synuclein, DJ-1 or LRRK2 (Langston and Ballard, 1983; Schapira et al., 1990; Hsu et al., 2000; Silvestri et al., 2005; Martin et al., 2006; Junn et al., 2009; Angeles et al., 2011; Mena et al., 2011), results in decreased electron transport chain activity [1] and the ensuing ATP synthesis decrease and ROS increase [2]. Decreased ATP levels impairs ISC synthesis that results in decreased activity of ISC-containing proteins and increased mRNA binding activity of the iron homeostasis protein IRP1. IRP1 activation leads to increased DMT1 and TfR1 expression (Lee et al., 2009; Mena et al., 2011) [3] and the ensuing iron accumulation (Asenjo, 1968; Dexter et al., 1987; Faucheux et al., 2003; Michaeli et al., 2007) [4]. Increased ROS and increased redox-active iron promotes the consumption of intracellular reductants such as GSH and ascorbate (Perry et al., 1982; Ehrhart and Zeevalk, 2003; Núñez et al., 2004; Jomova et al., 2010) [5], resulting in a further decrease in mitochondrial activity and ISC synthesis (Harley et al., 1993; Gu et al., 1998b; Jha et al., 2000; Chinta et al., 2007; Danielson et al., 2011). Another input to this cycle is contributed by inflammatory cytokines liberated by activated microglia and astrocytes (Mogi et al., 1994) [6], which enhance mitochondrial dysfunction (Tatsumi et al., 2000; Xie et al., 2004; Hunter et al., 2007; Djafarzadeh et al., 2011) [7], increase ROS production (Grzybicki et al., 1996) [8] and increase iron accumulation by modifying the expression of the iron transporters DMT1 and FPN1 (Urrutia et al., 2013; Wang et al., 2013) [9]. As discussed in the text, increased ROS back-feed the production of cytokines. Increased ROS levels, in particular increased hydroxyl radical generation, produces increased oxidative damage, which is counteracted by antioxidant defenses [10]. In time, the positive feedback loop of mitochondrial dysfunction, iron dyshomeostasis and inflammation results in alpha-synuclein aggregation, proteasomal dysfunction, changes in mitochondrial fission/fusion dynamics, opening of the mitochondrion PTP, increased cytoplasmic cytochrome c and activation of death pathways [11]. Debris and toxins from dying neurons enhance the activation of glial cells, which contributes to the inflammatory network (Zecca et al., 2008; Hirsch and Hunot, 2009; Gao et al., 2011) [12].

In summary, because of the innate interconnectivity of mitochondrial complex I dysfunction, iron accumulation, oxidative stress, and inflammation, probably the initiation of any one of these factors will induce or enhance the others through the generation of a positive feedback loop that in time will end in apoptotic neuronal death. Still unanswered is the question of why neurons of the SNc are so particularly prone to carry-on this cycle. On examination of this cycle, several therapeutic targets come to mind. Its intervention should result in prolonged life of the affected neurons.

## Conflict of Interest Statement

The authors declare that the research was conducted in the absence of any commercial or financial relationships that could be construed as a potential conflict of interest.
